# Medical Electricity

**Published:** 1873-12

**Authors:** 


### Medical Electricity.

Messrs. Editors,—With your permission I desire to say a few words by way of reply to a communication on the subject of medical electricity of your correspondent, Rusticus, who seems equally anxious to get information himself and to impart it to others. Electricity is a science of vast depth and intricacy, and, more especially, that part of it which belongs to animal structures; in proof of which I will mention that even Carpenter, accustomed to those profound researches we admire in his Physiology, confesses himself unable to follow Du Bois-Reymond to the full extent of those investigations he has made on this subject. Difficult, indeed, would be the practice of medical electricity, and confined to the hands of very few, if one had to go to the very bottom of inquiries like these, and might not venture, for example, to send a current from his battery through a nerve until he had first considered how this artificial influence would affect certain numberless electrical circles moving spontaneously within the infinitesimal molecules of the part itself, each pair of which becomes peripolar or depolar, as the nerve is in a state of action or at rest. I question, indeed, whether even those who amuse themselves with such minute speculations, bring them into actual practice, or are not rather guided by those external symptoms, and that ordinary experience, equally within the reach of my friend Rusticus and the great multitude of other practitioners.

*Haud inexpertus loquor.* When, some years since, my attention was first directed to electricity as a means of curing disease, the instruments themselves, I confess, presented a difficulty at the very outset; they were new to me, and, as a matter of consequence, not understood. I then had recourse to books, which began with abstract, half metaphysical discussion, extending far into the volume before any practical matter was approached. This mass of introductory matter I do not, by any means, mean to say was useless; I would only assert that it was too much labored and remote for a beginner, and that all the essential points are comparatively simple, and such as may be mastered without any unusual share of difficulty. Since this time, many good works on the subject have been written, as those of Althaus, Reynolds, Tibbits, Hamilton, Meyer, etc., and many admirable machines constructed, as those of Remak, Fromhold, Meyer, and the Galvano-Faradic Manufacturing Co. of New York; the latter are those I now use, as being at once simple and efficacious.

While making these remarks, I am not at all to be understood as if desirous of defending those itinerant and other electricians whom Rusticus so justly decries; on the contrary, such ignorant pretenders deserve no countenance, and, as in the instance of the lady with her ' primary" and "secondary" who brought on hemiplegia, should be prosecuted and punished by law. All I would advance, is, that the practice of electricity is open to every physician; that the success with which he uses it will depend mainly on his knowledge of disease, and that there is no secret in this, any more than in any other department of medical science. While I would caution the public against the quack, local, itinerant, male and female, I would also remind the physician of his own ability, and encourage him to make use of electricity himself, if for no other motive than to take it out of the hands of the uneducated. It would, indeed, be a strange thing to see the country overrun with imposters who carry a "box" filled with mischief, like that of Pandora, while a medical man is obliged to look on, or send his patient to a specialist in Boston, New York, or elsewhere.

But I fear, gentlemen, that I have taken up too much of your valuable space, and must conclude somewhat abruptly by advising Rusticus, and others who desire to secure to their patients the benefits of electricity, to procure some such instructive books and some such effective instruments as those above spoken of, when, in a short time, with a little study and a little practice, they may thrust out the empiric, vindicate their own claims, and not trouble the specialist. I have the honor to be yours,

—*Boston Med. and Surg. Journal.* URBANUS.

